# Artificial intelligence, epistemic authority, and emerging risks in veterinary clinical decision-making

**DOI:** 10.3389/fvets.2026.1861384

**Published:** 2026-07-15

**Authors:** Abdullah Eryol

**Affiliations:** Department of Veterinary History and Deontology, Faculty of Veterinary Medicine, Atatürk University, Erzurum, Türkiye

**Keywords:** artificial intelligence, authority delegation, epistemic authority, explainability, professional judgment, veterinary clinical decision-making

## Abstract

Artificial intelligence (AI) is becoming increasingly visible in veterinary medicine, not only in diagnostic and decision-support applications but also in ways that may influence how clinical reasoning is structured. Because veterinary clinical decision-making is shaped by animal welfare, owner preferences, economic constraints, and legal ambiguity, AI should be evaluated not only in terms of performance, but also in relation to epistemic authority and professional judgment. This article is a theoretical narrative review based on targeted searches in PubMed, Scopus, and Google Scholar. The literature was examined conceptually with particular attention to clinical decision-making, explainability, automation bias, epistemic authority, and veterinary ethics. This review identifies two theoretically plausible areas of risk that may arise under certain conditions and require empirical testing in veterinary clinical settings. The first is potential authority delegation, in which AI outputs may gradually become de facto reference points that guide clinicians’ reasoning and narrow the space for independent judgment. The second is potential epistemic-normative reshaping, whereby AI may indirectly influence the informational, evaluative, and justificatory framework of clinical decisions, particularly in legally uncertain and ethically contested areas such as euthanasia, off-label use, and unlicensed treatments. In veterinary medicine, the central question is not only whether AI is accurate, but what kind of position it occupies within clinical reasoning. Given these potential risks, AI should be treated as a bounded and critically reviewable support tool rather than as an epistemic authority.

## 1 Introduction

Artificial intelligence (AI) is becoming increasingly visible across a broad range of applications in veterinary medicine, extending from diagnostic imaging and clinical decision support systems to biomedical research and educational processes (1–3). In particular, large language models (LLMs) and generative systems based on them have begun to be incorporated more directly into veterinary clinical practice through functions such as summarizing clinical records, structuring symptoms, listing possible diagnoses, and suggesting additional questions that may guide the diagnostic process (4). The fact that machine learning-based systems have achieved high levels of accuracy in some areas (5) and, in certain tasks, can perform at a level comparable to novice clinicians, or even more consistently at times (6), makes it necessary to consider AI not merely as an auxiliary tool, but as a factor that may influence the formation of clinical judgment.

However, the process of clinical decision-making in veterinary medicine has a multilayered structure that cannot be reduced solely to the interpretation of biological data (7). Veterinarians shape their decisions not only according to the medical characteristics of the disease, but also within the framework of owner preferences, economic constraints, animal welfare considerations, and, in some cases, uncertain or fragmented normative regulations (8, 9). For this reason, the integration of AI into veterinary clinical practice should be evaluated not only in terms of accuracy, efficiency, or technical performance (10–12), but also through questions concerning how clinical reasoning is constructed, which forms of knowledge are regarded as more legitimate, and in whom decision-making authority is concentrated (13). This concern is further illustrated by cross-domain evidence from professional chess, a field in which highly capable AI has already transformed the social organization of expertise. Anicker (14) analysis shows that AI did not merely improve analytical performance in chess, but also reshaped the meaning of the practice, redistributed authority, reshaped power relations, and created new social roles. Although chess differs from veterinary medicine because it is a rule-bound and low-stakes domain, it provides a model case for how sustained AI superiority may transform the epistemic order of a professional practice by changing who or what is treated as the authoritative judge of performance (14). Especially in the case of black-box systems, the coexistence of high performance (5, 6) and limited explainability (15) makes the question of what kind of epistemic status AI will acquire in clinical decision-making even more significant.

Although the existing literature offers a growing discussion on the areas of use, ethical risks, and technical limitations of AI in veterinary medicine (15–18), the possibility that these systems may acquire a function similar to epistemic authority in veterinary clinical decision-making processes has so far received only limited attention (13). Yet even if AI systems are not theoretically regarded as epistemic authorities in a direct sense, it has been argued that, in practice, they may come to function as reference points that shape the reasoning of human experts because of perceptions of high performance, trust in automation, and increasing dependence on them in clinical decision-making processes (19, 20). If such a risk arises in veterinary medicine, it may take a distinct form because of the structural characteristics of veterinary clinical decision-making. This narrative review aims to discuss the increasing role of AI in clinical decision-making processes in veterinary medicine, focusing on epistemic authority, explainability, and professional judgment.

## 2 Review method

This article was designed as a theoretical narrative review rather than a systematic review. The literature was searched in PubMed, Scopus, and Google Scholar using keywords and keyword combinations related to *“artificial intelligence,” “veterinary medicine,” “clinical decision-making,” “epistemic authority,” “explainability,” “automation bias,” “black-box AI,” “authority delegation,” “professional judgment,” “off-label use,” “euthanasia,” and “veterinary ethics.”*

The literature search was last updated in May 2026. Sources were included when they addressed at least one of the following areas: AI applications in veterinary practice; clinical decision-making; explainability and trust in medical AI; epistemic authority and automation bias; or ethically and legally contested veterinary decisions. Human-medicine and general AI ethics sources were included only when they provided conceptual tools relevant to the veterinary context. They were not treated as direct empirical evidence for veterinary practice. Because the purpose of the review was conceptual rather than systematic, no formal exclusion protocol or risk-of-bias assessment was applied. Instead, sources were screened for conceptual relevance to the review question. Given the limited and heterogeneous nature of the available veterinary evidence, a systematic synthesis was not considered appropriate for the aims of this paper. The article therefore focused on developing a conceptual framework.

In addition to peer-reviewed journal articles, a limited number of emerging or non-peer-reviewed sources were included when they were directly relevant to the conceptual argument and when no equivalent peer-reviewed veterinary source was available. These sources were used cautiously and were not treated as primary empirical evidence for the existence or prevalence of AI-related epistemic risks in veterinary medicine. One non-peer-reviewed source was used to document the reported use of GS-441524 in Türkiye as an example of a legally and ethically contested veterinary treatment context. The concepts of *“System 3”* and *“cognitive surrender”* were initially drawn from Shaw and Nave (21) emerging tri-system framework and were treated in this review as conceptual tools rather than as established veterinary evidence. Their relevance was further supported by a recent peer-reviewed discussion of agentic AI, System 3, and cognitive surrender in medical imaging education. Pivotal sources were selected for Table 1 when they directly supported one of the review’s main claims, and their evidential status and limitations are explicitly indicated in the table.

**Table 1:** Evidence base for the main claims of the review.

Reference	Study type	Main contribution	Species/setting	Sample size	Comparator/control	Outcome measure	Key limitation
Gray and Fordyce (7)	Legal/ethical commentary	Shows that veterinary decision-making differs from human medicine and shares similarities with pediatric medicine because decisions are mediated through the veterinarian–animal–owner	Companion animal medicine	N/A	N/A	Best-interests decision-making	Not AI-specific
Brown et al. (9)	Conceptual framework	Provides the spectrum-of-care framework, showing that veterinary decisions are shaped by cost, technology, skill, and available resources	Clinical veterinary practice	N/A	N/A	Spectrum of care framework	Normative framework; not AI-specific
Kipperman et al. (8)	Cross-sectional survey	Supports the claim that economic constraints influence small animal veterinarians’ decisions and patient care	Small animal practice, USA and Canada	1,122 veterinarians	No formal control	Opinions and actions regarding cost of care	Self-report; small animal focus; not AI-specific
Janke et al. (23)	Qualitative focus group study	Supports the claim that owner–veterinarian communication and information exchange shape clinical decision-making	Companion animal practice	27 owners; 24 veterinarians	Owner and veterinarian focus groups	Perceptions of information exchange and clinical decision-making	Qualitative; context-specific; not AI-specific
Coghlan and Quinn (15)	Conceptual ethics review	Provides a veterinary ethics basis for treating AI as a support tool rather than as a replacement for professional judgment	Veterinary medicine	N/A	N/A	Ethical analysis of AI use in veterinary medicine	Broad conceptual review; limited empirical basis
Cohen and Gordon (16)	Ethical/legal review	Shows that veterinary AI/ML raises ethical and legal concerns in specialist clinical contexts	Veterinary radiology and radiation oncology	N/A	N/A	Ethical and legal analysis	Specialty-specific; not general clinical decision-making
London (10)	Conceptual analysis	Supports the distinction between technical accuracy and explainability in medical AI	Medical AI	N/A	N/A	Accuracy versus explainability	Human-medicine context; transfer to veterinary medicine is inferential
Durán and Jongsma (11)	Philosophical/ethical analysis	Provides a framework for justified trust in black-box medical AI under validation and oversight conditions	Medical AI	N/A	N/A	Conditions for justified trust in black-box AI	Not veterinary-specific
Kerasidou and Kerasidou (13)	Philosophical analysis	Supports the claim that AI performance should not be conflated with epistemic authority	Medical AI / clinical practice	N/A	N/A	Epistemological differences between AI systems and clinicians	Theoretical; not veterinary-specific
Okur et al. (6)	Comparative performance study	Provides veterinary evidence that LLMs may perform competently in specific decision-support scenarios	Veterinary theriogenology scenarios	15 scenarios; 2 expert clinicians; 2 novice veterinarians; 2 LLMs	LLMs compared with expert clinicians and novices	5-point global quality score assessed by blinded expert panel	Simulated scenarios; not real clinical outcomes
Okur et al. (39)	Retrospective diagnostic performance study	Provides veterinary evidence that deep-learning imaging models can support automated image-based diagnosis	Dogs / retinal detachment detection from fundus photographs	275 dogs; 2,000 fundus photographs	Comparison of pretrained CNN architectures	Accuracy, precision, recall, F1-score, and AUC	Single imaging task; prospective external validation needed
Milella and Cabitza (33)	User study	Supports the claim that users may attribute epistemic authority to AI systems	General AI users	610 participants	No clinical or veterinary comparator	Attribution of epistemic authority / trust in AI	Not veterinary-specific; transfer to clinical settings is inferential
Branda and Ciccozzi (19)	Conceptual analysis	Supports the concern that automation may encourage authority delegation and weaken cognitive sovereignty	AI-driven life sciences	N/A	N/A	Cognitive sovereignty and automation	Not veterinary-specific
Shaw and Nave (20)	Three preregistered behavioral experiments with within-paper trial-level synthesis	Provides the emerging concepts of cognitive surrender and System 3-like influence on human reasoning	Human–AI interaction	1,372 participants; 9,593 trials	Brain-Only vs. AI-Assisted; AI-Accurate vs. AI-Faulty output	Accuracy, AI consultation, follow/override behavior, confidence	Not veterinary-specific; reasoning-task setting rather than clinical decision-making; appears to be an emerging/preprint framework unless peer-reviewed status is confirmed
Reagan et al. (40)	Cross-sectional survey	Supports the claim that veterinary students expect AI/ML to become part of veterinary medicine despite limited AI literacy	Veterinary students	176 students	No formal control	Self-reported AI/ML knowledge, curriculum exposure, interest in learning, expected future use	Single-institution survey; self-report; educational context rather than clinical decision-making
Tarillion et al. (47)	Quantitative cross-sectional survey	Supports the claim that off-label use illustrates regulatory gray zones in small animal practice	Small animal medicine / Germany	358 participants	No formal control	Frequency, reasons, and patterns of off-label antibiotic use	Single-country survey; self-report; focused on antibiotics
Gokalsing et al. (49)	Systematic review	Supports the claim that clinically promising but legally contested treatments can complicate veterinary decision-making	Cats / FIP / GS-441524	11 studies; 650 FIP cases	Across included studies	Treatment success rate and clinical outcomes	Heterogeneity across included studies; regulatory status varies by country

## 3 Veterinary clinical decision-making

Clinical decision-making in veterinary medicine, unlike the classical physician–patient relationship in human medicine (22), takes shape within a triadic relationship established among the veterinarian, the animal, and the animal owner (7, 15). For this reason, clinical decision-making in veterinary medicine is regarded as a relational process in which different interests, expectations, and constraints intersect. Although the animal is the patient directly affected by the intervention, it is not a subject capable of verbally expressing its preferences or making decisions on its own behalf. Therefore, the choice of treatment is determined by the animal owner’s expectations, economic capacity, and limits of acceptance regarding treatment (8, 9, 23). Within this multilayered structure, the epistemic role of the veterinarian extends beyond that of a mere diagnostic expert (24); it takes on the character of a reasoning authority that weighs together different types of knowledge, conflicts of interest, and normative justifications (7, 25, 26). In this context, although the process of veterinary clinical decision-making bears partial similarities to human medical contexts involving pediatric or legally incapacitated patients, in that decision-making authority does not lie directly with the patient (7), it also differs from them because of the distinct ethical and legal status of the animal. Indeed, studies focusing on the veterinarian-patient-owner interaction show that clinical decision-making is one of the most complex dimensions of this relationship, that communication style influences owners’ participation in the decision-making process, and that many owners expect a more collaborative decision-making process with the veterinarian (23, 27).

Another epistemically distinctive aspect of veterinary medicine is that economic constraints directly shape both the content and the feasibility of clinical decisions. Because the cost of veterinary care is, in most cases, borne directly by the animal owner, financial limitations play a decisive role in the clinical decision-making process (28, 29). In particular, the high cost of advanced veterinary care may render certain diagnostic and therapeutic options inaccessible for many patients (29). Kipperman et al. (8) showed that the cost of care is one of the main factors affecting veterinarians’ views, practices, and decisions regarding patient care in small animal medicine. Similarly, studies on access to veterinary care have demonstrated that financial constraints make it more difficult for animal owners to obtain care services (28, 30). The *“spectrum of care”* approach developed by Brown et al. (9) conceptualizes veterinary care not as a single ideal standard of treatment, but as a range of acceptable options that vary according to cost, technology, skill, and available resources. These findings suggest that economic constraints in veterinary medicine shape the content of clinical decisions. Accordingly, the veterinarian’s decision is based not only on identifying the most appropriate option from a biomedical perspective, but also on evaluating which option is feasible under existing economic conditions (Table 2).

**Table 2:** AI system types and corresponding epistemic risks in veterinary clinical decision-making.

AI system type	Typical output	Main clinical use	Most relevant epistemic risk	Less central risk
Machine-learning diagnostic tools	Risk score, probability, classification	Diagnosis, prognosis, triage	Overreliance on model probability; dataset bias; poor generalization	LLM-style hallucination
Deep-learning imaging models	Image label, lesion detection, probability map	Radiology, ophthalmology, pathology, dermatology	Visual epistemic deference; reduced independent image interpretation; domain shift	Normative reshaping
Clinical decision support systems	Alerts, recommendations, ranked options	Treatment planning, diagnosis, triage, protocol-based decision-making	Automation bias; default-option effect; authority delegation	Free-text hallucination
Large language models	Narrative answer, differential list, explanation, owner communication text	Information synthesis, clinical reasoning support, documentation, communication	Plausible but unsupported reasoning; hallucination; cognitive surrender; normative framing	Image-specific error

The table is intended as a conceptual synthesis rather than an empirical validation of risk magnitude. The risks listed should not be interpreted as applying equally or with the same degree of empirical support across all AI systems.

## 4 Epistemic authority in clinical practice

Clinical decision-making is regarded as a rational process that requires the joint consideration of factors such as knowledge, reasoning, awareness of bias, critical thinking, communication, appropriate use of tests, and patient preferences. Within this framework, it is emphasized that the patient should also be involved in clinical decisions made about their care (31). This indicates that the process of clinical decision-making has not only a technical but also a relational dimension. Indeed, in Emanuel and Emanuel’s discussion of different approaches to the physician–patient relationship, clinical decision-making is shown not to be a fixed and one-directional structure, but rather one that is shaped in different ways depending on the nature of the relationship. In particular, in the deliberative model, the physician is positioned not merely as a *“technician”* who provides information, but also as an actor who discusses the patient’s health-related values and guides the shaping of the decision-making process in light of those values (24). Popowicz explains the physician’s informational privilege through the concept of *“epistemic authority.”* However, epistemic authority here does not mean that the physician is an authority who dictates to the patient what to do or makes decisions unilaterally. Rather, it refers to the physician’s ability, by virtue of their knowledge, experience, and methodological competence, to structure the clinical decision-making process on a scientific and rational basis, to justify that process, and to support the patient’s reasoning within that framework. For this reason, the physician is understood not simply as the person who *“knows best,”* but as an actor who makes decisions together with the patient and respects the patient’s autonomy (26). In veterinary medicine, however, this structure becomes more complex. As noted earlier, the veterinarian must take into account not only the patient’s condition but also the animal owner’s expectations, economic constraints, and the sustainability of care. Indeed, findings from a qualitative study indicate that veterinarians do not always follow a systematic evidence-based approach in their clinical decision-making processes. In some situations, veterinarians appear to rely on previous experience, seek the opinions of colleagues, or turn to more practical decision-making pathways (32). At this point, it may be argued that the process of clinical decision-making in veterinary medicine is more clearly shaped by contextual factors than the understanding of epistemic authority discussed in human medicine (Table 3).

**Table 3:** Conceptual mapping of AI-related epistemic risks across veterinary clinical decision points.

Veterinary decision point	Typical AI output	Why AI may influence reasoning at this point	Main risk	Safeguard
Triage	Risk score; urgency ranking	Early AI ranking may anchor the perceived urgency of the case and shape subsequent reasoning	Automation bias; authority delegation	Require clinician justification of urgency independent of AI output
Diagnosis	Differential diagnosis list; image label; probability ranking	Ranked outputs may narrow the diagnostic search space and make non-listed differentials less visible	Epistemic deference; cognitive surrender	Require an independent differential list before AI consultation
Prognosis	Survival estimate; risk prediction; expected treatment response	Numeric estimates may appear more objective and certain than the underlying evidence allows	Overreliance on probabilistic output	Interpret prediction together with uncertainty, comorbidities, welfare, owner capacity, and clinical context
Treatment selection	Ranked treatment options; protocol suggestion	AI may make one option appear more standard, defensible, or clinically safer	Authority delegation	Require clinical, welfare-related, financial, and legal justification beyond AI ranking.
Off-label or unlicensed treatment	Literature summary; risk–benefit framing; alternative treatment suggestions	AI may pre-frame a legally uncertain option as either clinically reasonable or professionally unacceptable	Epistemic-normative reshaping	Keep AI informational only; require explicit veterinary responsibility for legal and professional judgment
Euthanasia discussion	Quality-of-life summary; communication script; option framing	AI-generated language may frame euthanasia as more or less acceptable and influence owner deliberation	Normative narrowing; persuasive framing	Separate biomedical facts, welfare assessment, owner values, and ethical judgment
Client communication	Consent text; owner explanation; discharge instructions	Fluent AI text may appear neutral while embedding assumptions, priorities, or persuasive framing	Persuasive artificial authority	Veterinarian must review, contextualize, and personalize all AI-generated communication
Record summarization	Summarized history; diagnostic results; previous treatments	Summaries may omit uncertainty, conflicting observations, owner constraints, or clinically relevant detail	Information filtering; omission bias	Original records must remain available; AI summaries must not replace full clinical review

This table is intended to identify plausible decision points in veterinary clinical practice at which the epistemic risks discussed in this review may arise. It does not claim that these risks are empirically prevalent or causally established; rather, it provides a conceptual mapping to clarify where such risks may become relevant in clinical reasoning.

## 5 AI system types, outputs, and risk profiles

**Figure 1:**
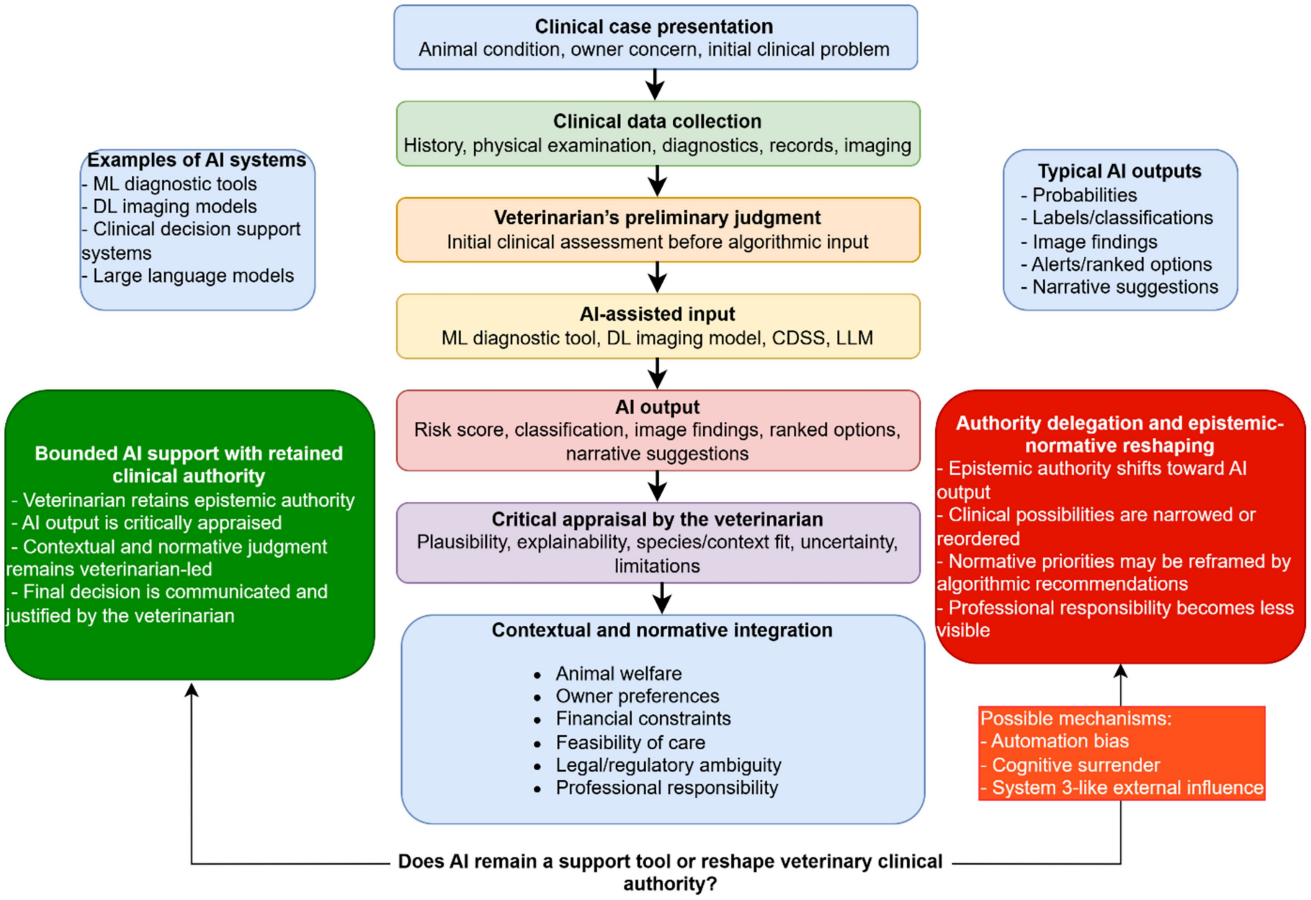
Clinical AI decision-making pathway in veterinary practice. The figure shows how AI-assisted inputs enter the clinical reasoning process following case presentation, data collection, and the veterinarian’s initial assessment. When AI outputs are critically evaluated and interpreted within the broader clinical context, they may function as a bounded decision-support tool. However, when such outputs are insufficiently scrutinized, epistemic authority may gradually shift toward the AI system, contributing to epistemic-normative reshaping. In this process, clinical possibilities, priorities, and value-laden considerations may be narrowed, reordered, or reframed through algorithmic recommendations. Automation bias, cognitive surrender, and System 3-like external influence are presented as potential mechanisms underlying this shift.

AI does not refer to a single type of technology. Machine-learning diagnostic tools generally use structured clinical, laboratory or patient-level data to generate risk estimates, classifications or diagnostic probabilities. Deep-learning imaging models usually analyze visual data such as radiographs, ultrasound images, retinal images, histopathology slides or dermatological images and produce labels, detections, probability maps or segmentation outputs. Clinical decision support systems combine clinical variables, rules, guidelines, prediction models, or algorithmic recommendations to support triage, diagnosis, treatment planning, or monitoring. LLMs differ from these systems because they operate primarily through text: they may summarize records, generate differential diagnosis lists, suggest questions, draft explanations or produce owner-facing communication. These systems therefore differ in technical architecture and in the kinds of epistemic influence they may exert on clinical reasoning (Figure 1).

AI systems used or proposed in clinical settings differ substantially in their data inputs, output formats, explainability, error profiles, and clinical functions. Machine-learning diagnostic tools, deep-learning imaging models, clinical decision support systems, and large language models therefore should not be evaluated under a single undifferentiated risk category. The following table provides a conceptual mapping of these system types and the epistemic risks most commonly associated with each, drawing on literature concerning AI applications, medical AI explainability, epistemic authority, automation bias, and cognitive surrender (1, 4, 10, 11, 13, 15, 16, 19–21, 33, 34).

## 6 AI, explainability, and the limits of machine authority

The limited transparency many AI systems provide regarding how their outputs are generated, especially in the case of deep learning-based models, constitutes an important area of debate in their clinical use. Chan argues that even when the outputs of *“black-box”* medical AI algorithms are reliable, the inability to explain decisions to patients may undermine appropriate and respectful patient care (35). By contrast, London maintains that not every decision in clinical practice rests on a full causal explanation, and that in some cases epistemic value may depend less on process transparency than on reliable performance validated across different conditions (10). Similarly, Durán and Jongsma (11) and Ferrario (36) show that systems whose internal mechanisms are not fully transparent may still be regarded as reliable to a certain extent, provided that they undergo adequate testing, validation, and oversight processes.

However, it is precisely at this point that a critical boundary emerges. AI’s success in producing reliable outputs should not be conflated with epistemic authority. As Kerasidou and Kerasidou (13) emphasize, epistemic authority in medicine rests not merely on statistical pattern recognition, but on a particular scientific method, theoretical understanding, and practice of clinical justification. For this reason, although AI can make important epistemic contributions to clinical decision-making, it cannot by itself assume the higher-order reasoning function of evaluating why a particular outcome should be preferred, under what conditions it is limited, and under what responsibility it should be used. Accordingly, the value of AI in veterinary clinical practice lies not in treating it as a machine authority in its own right, but in positioning it as a supportive tool with clearly defined boundaries within the reflective reasoning of the human expert (13). Similarly, both the human and veterinary medical literature emphasize that AI should be positioned not as a decision-maker in clinical practice, but as a decision-support tool (13, 15, 18, 35, 37). However, Milella and Cabitza (33) show that individuals may attribute epistemic authority to AI systems, and that this tendency is particularly associated with trust in automation and perceived performance. It has been reported that the embedding of AI-based systems in decision-making processes carries risks such as automation bias, epistemic delegation, and the erosion of professional skill, and for this reason the concept of *“cognitive sovereignty”* has gained increasing importance (19). In a similar vein, Shaw and Nave (20) *“Tri-System Theory”* proposes that an external and artificial third system, namely *“System 3,”* has been added to the classical dual-process model of cognition; in this view, AI no longer functions merely as an auxiliary tool, but in some situations as a cognitive agent that supports, restructures, and may even replace the reasoning process. Within this framework, the authors describe people’s tendency to adopt AI-generated responses without sufficiently questioning them, especially when faced with fluent, rapid, and confident outputs as *“cognitive surrender.”* Their experimental findings show that when AI is correct, performance improves, but when it is wrong, individuals may suspend their own reasoning and nonetheless tend to follow the erroneous output. Moreover, the fact that access to AI can increase confidence even in cases where the output is incorrect indicates that the issue is not merely one of accuracy, but also of confidence, control, and the question of to whom reasoning ultimately belongs (20).

This suggests that AI tools may do more than merely provide decision support; through repeated use, perceived high performance, and user trust, they may come to function as de facto reference points and, over time, as forms of epistemic authority. Taken together, these findings indicate that the issue is not simply whether AI produces correct results, but how it becomes embedded in the reasoning process, to what extent it narrows alternative ways of thinking, under what conditions it weakens critical oversight, and to what extent it gradually produces the effect of a de facto authority.

## 7 Artificial intelligence risks in veterinary medicine

### 7.1 Risk 1: authority delegation

In this review, the concepts of *“authority delegation”* and *“cognitive surrender,”* developed in human medicine and general human–AI interaction, are used as analytical tools to examine whether comparable processes may also arise in veterinary clinical decision-making. Their application to veterinary medicine is therefore theoretical and requires empirical testing. Authority delegation refers to the possibility that AI may cease to remain merely a technical support tool and instead become a de facto reference center that guides the clinician’s reasoning, narrows the horizon of decision-making, and shapes which option is regarded as more reasonable, reliable, or defensible (19). Cognitive surrender, in turn, explains how this shift becomes possible not only at the institutional or normative level, but also at the cognitive level: the clinician may begin to treat the AI output not as a recommendation that should be subjected to critical evaluation, but increasingly as the closest approximation to the default truth, and as a safe and ready reference point (20). Thus, even under conditions in which the decision appears to continue being made by a human, the epistemic center of gravity of reasoning may quietly shift toward algorithmic outputs. This framework may have particular explanatory relevance to veterinary medicine. As noted above, veterinary clinical decision-making is not a process that can be defined solely by technical criteria such as diagnostic accuracy or treatment efficacy; rather, it requires the simultaneous consideration of the animal’s welfare, the reduction of suffering, prognosis, owner preferences, economic possibilities, sustainability of care, professional responsibility, and at times legal uncertainties (8, 9, 23, 28, 38). For this reason, the *“most correct decision”* in veterinary medicine often does not simply mean the biomedically strongest option; it also involves a normative judgment concerning which option is justifiable, defensible, and feasible in a given concrete situation. Precisely for this reason, the incorporation of AI outputs into clinical decisions in veterinary medicine is not merely a matter of technical assistive technology; it is a question of where the normative center of reasoning is to be located. Delegation defines the risk that authority and decisional weight may shift from the clinician to the algorithmic output, whereas cognitive surrender explains how this shift becomes possible within the cognitive functioning of the subject. In clinical practice, when the veterinarian begins by treating the recommendation offered by AI merely as *“additional information,”* but over time comes to regard it as the default correct answer, the first option to consult, or a safe reference point, the ground for delegation is established through cognitive surrender.

The particular weight of this risk in veterinary medicine also arises from the representational nature of decision-making. Whereas in human medicine the decision is structured, at least in the ideal-typical case, around patient autonomy and the patient’s own values, in veterinary medicine the patient cannot verbally express its preferences. The decision is made on behalf of the animal, but most often within a representational relationship conducted with the owner (7, 15). Accordingly, the clinician’s reasoning depends not only on scientific knowledge, but also on the ability to interpret the animal’s interests within the framework of owner demands, care relationships, and practical constraints (9). When this interpretive function is weakened, AI output does more than merely offer a technical recommendation; it may also assume a role that narrows the normative space and pre-frames which options are worth considering. The danger here is not that AI is explicitly declared a *“decision-maker.”* The real danger is that, through repeated use, fluency, speed, the promise of standardization, and the perception of high performance (4–6, 33, 39), AI may become the default starting point of clinical reasoning. In such a situation, the clinician may move away from being an autonomous reasoning subject who critically weighs algorithmic recommendations, while AI, though lacking formal authority, may begin to function as a de facto epistemic authority. For this reason, the problem is not merely that individual erroneous recommendations may be followed, but that the structure of reasoning itself, the way alternatives are generated, and the horizon of justification may increasingly become pre-shaped in algorithmic terms.

At this point, findings concerning veterinary students do not so much directly measure the risk of delegation as make visible its pedagogical and cognitive preconditions. Students are not wholly resistant to AI; some studies show that they believe these tools will play an important role in veterinary medicine in the future, find ChatGPT practical and relevant in certain contexts, and view it as open to professional use (40, 41). However, this relative openness is not supported by sufficient curriculum-based preparation that would enable them to critically evaluate the accuracy, reliability, limitations, and especially the authoritative mode of presentation of AI outputs. Indeed, one study shows that although students have low levels of knowledge about AI/ML, they nevertheless believe these technologies will be used in the future and express a demand for education in this area, while another study reveals that although students find traditional standardized-client-based communication training effective, they are more cautious about the educational value of AI-supported simulations and AI feedback (40, 42). The Australian study likewise shows that students may find ChatGPT practical and relevant, but regard its accuracy as problematic, and that a critically oriented task helps them recognize the limitations of such tools (41). This picture does not suggest that students explicitly declare AI to be an *“expert”;* rather, it suggests the existence of a pedagogical environment conducive to granting increasing epistemic weight to algorithmic outputs. Accordingly, the primary need in veterinary education is not merely to provide access to AI tools, but to develop a framework of critical AI literacy through which students can discuss under what conditions, to what extent, and on what grounds epistemic authority may be granted to algorithmic outputs (33).

### 7.2 Risk 2: epistemic-normative reshaping

Although epistemic-normative reshaping has not yet been directly tested as an empirical model in veterinary medicine, the existing literature makes it theoretically plausible to identify it as a potential area of risk. In this article, epistemic-normative reshaping refers to the possibility that AI systems may influence not only the information available to clinicians, but also how a clinical problem is framed, which forms of evidence are prioritized, which options appear reasonable, and which justifications come to be regarded as professionally or ethically defensible. This potential risk is especially relevant in areas of veterinary practice marked by legal, regulatory and ethical uncertainty.

In veterinary practice, some decisions are made not within clear and standardized legal boundaries, but within gray zones characterized by fragmented legislation, off-label practices, unlicensed yet clinically beneficial treatments, and situations in which the animal’s interests are represented only indirectly. Whereas in human medicine the termination of pregnancy is generally defined by clear legal limits and time restrictions, the normative framework governing such interventions in veterinary medicine appears more fragmented and open to interpretation (43, 44). Similarly, the absence of a common, stable, and clearly defined normative framework in veterinarians’ attitudes and practices regarding euthanasia further deepens the gray areas in this field (45).

One of the major legal constraints in veterinary ethics concerns the regulations governing the off-label use of medicines (46). Although the relevant legislation requires adherence to the authorized conditions of use for veterinary medicinal products, a recent study conducted in small animal practice in Germany shows that off-label antibiotic use is not an exceptional occurrence but a widespread clinical practice. This use arises for reasons such as the absence of an approved veterinary product, the perceived need to begin treatment before bacteriological test results are available, the clinical judgment that the licensed dose is insufficient, and practical difficulties in administration (47). Similarly, the more limited licensing processes for veterinary medicines compared with human medicines sometimes lead veterinarians to resort to off-label or unlicensed treatments in the interest of the animal’s welfare (47, 48). Indeed, according to the findings of a systematic review, the overall success rate in FIP cases treated with GS-441524 was reported as 84.6%, with the best outcomes generally obtained at doses of 5–10 mg/kg/day (49). Nevertheless, this drug remains unlicensed in many countries (49), including Türkiye, and despite the existing legal constraints, it has been reported to be used by veterinarians in Türkiye (50). These examples do not demonstrate epistemic-normative reshaping empirically, but they show the kinds of legally and ethically contested contexts in which such a risk could plausibly arise. They are not intended to suggest that epistemic-normative reshaping is already empirically established in off-label or unlicensed drug use. Rather, they illustrate the kinds of veterinary contexts in which AI-generated summaries, rankings, or recommendations may influence how clinical options are framed, compared, and justified. Precisely for this reason, even if AI can successfully detect biomedical patterns in such contexts, it cannot on its own determine which interest should take priority, which risk is acceptable, or which intervention may be regarded as defensible in a given situation. Nevertheless, by presenting the clinical problem within particular data categories, success metrics, and formats of recommendation, AI systems may indirectly frame the informational, evaluative, and justificatory grounds on which a decision is built. Moreover, the fact that AI ethics guidelines have largely been developed within an anthropocentric framework, and that animals are only minimally represented in these discussions, makes the normative foundation of veterinary medicine even more fragile (51). For this reason, the second risk that emerges here is not merely overreliance on algorithmic recommendations; rather, it is the unnoticed reshaping by AI of the informational, evaluative, and justificatory framework on which clinical reasoning is constructed in legally uncertain and ethically contested domains.

## 8 Conclusion and practical implications

In conclusion, AI should be considered in veterinary medicine not merely as a technical innovation that may increase diagnostic accuracy or reduce workload, but also as a potential epistemic factor that may influence the structure of clinical reasoning, the forms of justification employed, and the distribution of decision-making authority. Because veterinary clinical decision-making is shaped at the intersection of multiple axes such as the animal’s welfare, owner preferences, economic constraints, sustainability of care, and legal uncertainty, the integration of AI into this field cannot be grounded solely in terms of performance and reliability. This review identifies two theoretically plausible areas of risk in veterinary medicine that require empirical testing: potential authority delegation, in which AI outputs may become de facto reference points that guide clinicians’ reasoning, and potential epistemic-normative reshaping, whereby AI may influence the informational, evaluative, and justificatory foundations of clinical decisions in legally uncertain and ethically contested domains. For this reason, the central question in veterinary medicine is not whether AI produces correct results, but what kind of place it occupies within the order of clinical reasoning and to what extent it can become a de facto epistemic authority. Accordingly, the role of AI in veterinary clinical practice should be established not as an authority that replaces the reasoning of the human expert, but as a clearly bounded, critically reviewable, and normatively secondary support tool.

Within this framework, practical recommendations regarding AI in veterinary medicine should be structured around three axes indicated by the existing literature: preserving human oversight, strengthening critical AI literacy, and limiting the role of AI in normatively gray areas. First, AI systems should be defined in clinical practice not as independent decision-makers, but as tools that provide secondary support to human reasoning, because the literature emphasizes that responsibility for clinical and ethical justification must remain with the human agent (13, 15, 35). Second, veterinary education should incorporate a form of critical AI literacy through which students can question the accuracy, reliability, and persuasive mode of presentation of AI outputs, since existing studies show that students are open to these tools but have limited capacity for critical evaluation (40–42). Third, given that trust in automation and cognitive surrender may lead AI outputs to assume the status of de facto epistemic authority, reflexive re-justification steps should be incorporated into clinical use (19, 20, 33). Finally, in normatively contested areas such as euthanasia, off-label use, and unlicensed treatments, the role of AI should be kept at an auxiliary level, and it should be made explicit that the ethical and professional responsibility for the final decision remains with the clinician (46, 47, 51). This recommendation is consistent with the broader responsible-AI literature on meaningful human oversight. In clinical decision-making, human oversight should not be reduced to a formal *“human-in-the-loop”* step in which the clinician merely approves an AI-generated output. Rather, effective oversight requires sufficient competence, training, authority, and institutional support to question, override, and independently justify the use of AI outputs. In healthcare, this may require not only individual clinician vigilance, but also institutional governance structures capable of monitoring AI use, preventing automation bias, and preserving professional judgment (52).

### 8.1 Limitations

The main limitation of this study is that it offers a theoretical and narrative-based evaluation rather than a systematic review. For this reason, the selection of the literature was guided not by a comprehensive and reproducible search protocol, but by conceptual relevance. In addition, the risks discussed here, particularly authority delegation, epistemic-normative reshaping and cognitive surrender, have limited or no direct empirical support in veterinary clinical settings. Accordingly, the proposed framework should be regarded not as a set of validated conclusions, but rather as a conceptual point of departure for future empirical research.
